# Supplementary material for: Rapid Hormetic Responses of Photosystem II Photochemistry of Clary Sage to Cadmium Exposure
Source: Int J Mol Sci. 2020 Dec 22;22(1):41. doi: 10.3390/ijms22010041 (PMC7793146; doi:10.3390/ijms22010041)
Supplement: Supplementary file 1 [file ijms-22-00041-s001.pdf]

Rapid Hormetic Responses of Photosystem II Photochemistry of Clary Sage to Cadmium Exposure

Ioannis-Dimosthenis S. Adamakis, Ilektra Sperdouli, Anetta Hanć, Anelia Dobrikova, Emilia Apostolova and Michael Moustakas

Table S1. Definitions of the five main chlorophyll fluorescence parameters measured by the *Imaging PAM M-Series* system (*Heinz Walz Instruments*, Effeltrich, Germany)

Parameter	Definition	Measured
Fo	Minimum chlorophyll a fluorescence in the dark-adapted leaf, when the primary acceptor of PSII quinone A (QA) is maximally oxidized (PSII centers open)	Obtained by modulated measuring light of 0.5 μmol photons m^{-2} s^{-1}
Fm	Maximum chlorophyll a fluorescence in the dark-adapted leaf, when the primary acceptor of PSII quinone A (QA) is maximally reduced (PSII centers closed)	Obtained with a saturating pulse (SP) of 6000 μmol photons m^{-2} s^{-1}
Fs	Steady-state photosynthesis	Measured after 5 min illumination time before switching off the actinic light (AL) of 220 μmol photons m^{-2} s^{-1} or 900 μmol photons m^{-2} s^{-1}
Fo'	Maximum chlorophyll a fluorescence in the light-adapted leaf	It was computed by the Imaging Win software (Heinz Walz GmbH, Effeltrich, Germany) using the approximation of Oxborough and Baker (1997) Fo' =Fo/(Fv/Fm + Fo/Fm')
Fm'	Maximum chlorophyll a fluorescence in the light-adapted leaf	Measured with saturating pulses (SPs) every 20 s for 5 min after application of the actinic light (AL) of 220 μmol photons m^{-2} s^{-1} or 900 μmol photons m^{-2} s^{-1}

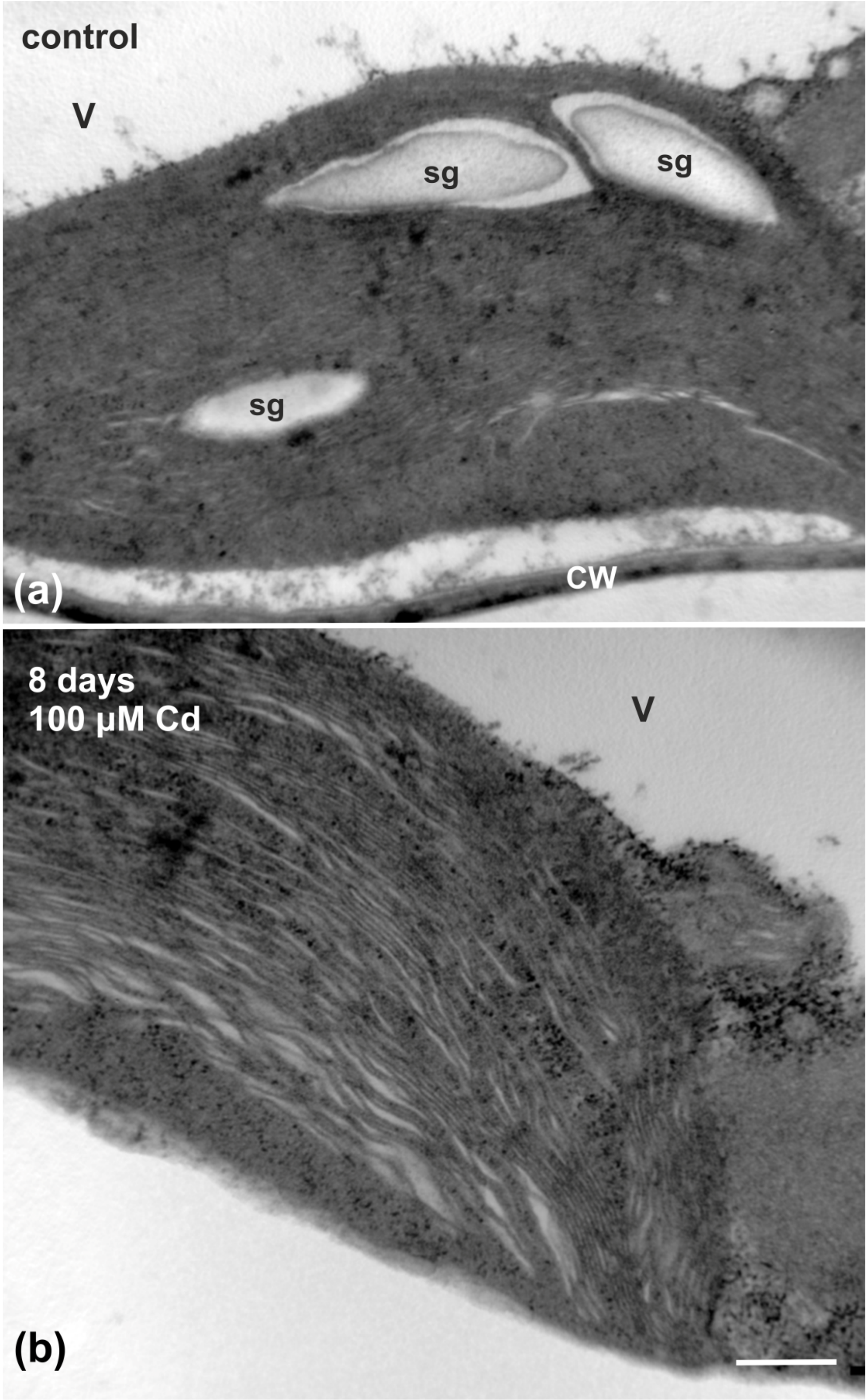

Figure S1. Transmission Electron Microscopy (TEM) images of control (untreated) chloroplasts (**a**) and 8-days Cd-treated **(b)** *Salvia sclarea* leaves. Chloroplasts appear electronically dense and upon Cd treatment with swollen thylakoids. **cw**: cell wall; **sg**: starch grain; **v**: vacuole. Scale bar: 500 nm.

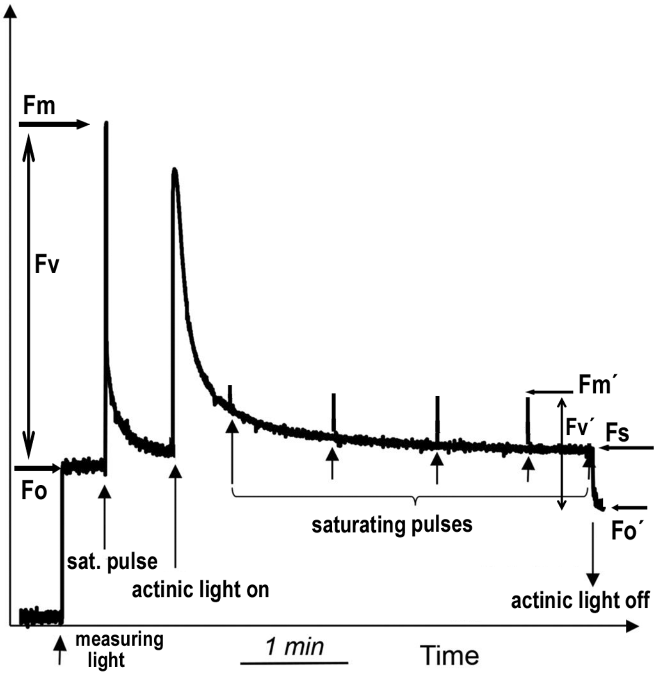

Figure S2. A typical modulated fluorescence trace of a dark-adapted leaf with F*o*, F*m*, F*o*′, F*m*′ and F*s* measurements. In the dark-adapted state a low intensity "measuring light" is switched on to elicit the minimal level of chlorophyll fluorescence, termed F*o*. A brief saturating pulse of light outcomes in the formation of the maximum yield of fluorescence, F*m*. The difference between F*m* and F*o* is the variable fluorescence, F*v*. The ratio F*v*/F*m* is the maximum quantum yield of PSII photochemistry. The application of saturating pulses under actinic light illumination closes all the reaction centers and provides the maximum fluorescence in the light-adapted state, termed F*m*′. The steady-state level of fluorescence in the light is termed, F*s* and is measured immediately before switching off the actinic light (Adopted from Moustakas et al. [*Plants* **2020,** 9, 962]).
